# A prospective randomised, open-labeled, trial comparing sirolimus-containing versus mTOR-inhibitor-free immunosuppression in patients undergoing liver transplantation for hepatocellular carcinoma

**DOI:** 10.1186/1471-2407-10-190

**Published:** 2010-05-11

**Authors:** Andreas A Schnitzbauer, Carl Zuelke, Christian Graeb, Justine Rochon, Itxarone Bilbao, Patrizia Burra, Koert P de Jong, Christophe Duvoux, Norman M Kneteman, Rene Adam, Wolf O Bechstein, Thomas Becker, Susanne Beckebaum, Olivier Chazouillères, Umberto Cillo, Michele Colledan, Fred Fändrich, Jean Gugenheim, Johann P Hauss, Michael Heise, Ernest Hidalgo, Neville Jamieson, Alfred Königsrainer, Philipp E Lamby, Jan P Lerut, Heikki Mäkisalo, Raimund Margreiter, Vincenzo Mazzaferro, Ingrid Mutzbauer, Gerd Otto, Georges-Philippe Pageaux, Antonio D Pinna, Jacques Pirenne, Magnus Rizell, Giorgio Rossi, Lionel Rostaing, Andre Roy, Victor Sanchez Turrion, Jan Schmidt, Roberto I Troisi, Bart van Hoek, Umberto Valente, Philippe Wolf, Heiner Wolters, Darius F Mirza, Tim Scholz, Rudolf Steininger, Gunnar Soderdahl, Simone I Strasser, Karl-Walter Jauch, Peter Neuhaus, Hans J Schlitt, Edward K Geissler

**Affiliations:** 1 University Hospital Regensburg, Department of Surgery, Regensburg, Germany; 2 Department of Surgery, Munich University - Grosshadern Campus, Munich, Germany; 3 Regensburg University Hospital, Center for Clinical Studies, Regensburg, Germany; 4 Department of Hepatobiliopancreatic Surgery and Transplants, Hospital Universitari Vall d'Hebron, Barcelona, Spain; 5 Gastroenterology Section, Department of Surgical and Gastroenterological Sciences, University Hospital of Padua, Padua, Italy; 6 Department of Surgery, Division of Hepato-Pancreatico-Biliary Surgery & Liver Transplantation, University Medical Center Groningen, University of Groningen, Groningen, the Netherlands; 7 Unité de Transplantation Hépatique, Service d'Hépato-Gastroentérologie, Hôpital Henri-Mondor, AP-HP, Université Paris-XII Val de Marne, Créteil, France; 8 Section of Hepatobiliary, Pancreatic, and Transplant Surgery, University of Alberta, Edmonton, Canada; 9 AP-HP Hôpital Paul Brousse, Centre Hépato-Biliaire, Villejuif, France, and Univ Paris-Sud, UMR-S776, Villejuif F-94800, France; 10 Department of General and Vascular Surgery, Johann Wolfgang Goethe University, Frankfurt, Germany; 11 Clinic for General-, Visceral- and Transplant Surgery, Medizinische Hochschule Hannover, Hannover, Germany; 12 Department of Gastroenterology and Department of General, Visceral and Transplantation Surgery, University Hospital Essen, University of Duisburg-Essen, Duisburg and Essen, Germany; 13 Service d'Hépatologie et Centre de Référence des Maladies Inflammatoires du Foie et des Voies Biliaires, Hôpital Saint-Antoine, Université Pierre et Marie Curie, Paris, France; 14 Unità di Chirurgia Epatobiliare e Trapianto Epatico, Dipartimento di Chirurgia Generale e Trapianti d'Organo, Azienda Ospedaliera di Padova, Padova, Italy; 15 Liver and Lung Transplantation Unit, Azienda Ospedaliera "Ospedali Riuniti", Bergamo, Italy; 16 Department of General and Cardiothoracic Surgery, University Hospital of Schleswig-Holstein, Campus Kiel, Kiel, Germany; 17 Service de Chirurgie Digestive et Transplantation Hépatique, Hôpital Archet, Nice, France; 18 Clinic for Visceral, Transplantation, Thoracic and Vascular Surgery, University Leipzig, Leipzig, Germany; 19 Department of General, Visceral and Vascular Surgery, University Hospital of the Friedrich-Schiller-University, Jena, Germany; 20 Scottish Liver Transplant Unit, The Royal Infirmary of Edinburgh, Edinburgh, EH16 4SA, UK; 21 Department of Surgery, Addenbrooke's Hospital, Cambridge, UK; 22 Department of General-, Visceral- and Transplantation Surgery, University Hospital Tübingen, Tübingen, Germany; 23 Department of Abdominal and Transplantation Surgery, Universite catholique de Louvain, Brussels, Belgium; 24 Clinic of Surgery, Department of Transplantation and Liver Surgery, Helsinki University Hospital, Helsinki, Finland; 25 Department of General and Transplant Surgery, Medical University Innsbruck, Innsbruck, Austria; 26 National Cancer Institute, Milan, Italy; 27 Department of Transplantation and Hepatobiliarypancreatic Surgery, Johannes Gutenberg University Mainz, Germany; 28 Liver Transplant Unit, Centre Hospitalier Universitaire (CHU) Saint Eloi, Montpellier, France; 29 Transplant, General and Emergency Surgery DPT, St. Orsola-Malpighi University Hospital, Bologna, Italy; 30 Abdominal Transplant Surgery, University Hospitals Leuven, Leuven, Belgium; 31 Department of Surgery, Transplantation and Liver Surgery Service, Sahlgrenska University Hospital, Gothenburg, Sweden; 32 Unità Operativa Chirurgia Generale e Trapianti di Fegato, Fondazione IRCCS Ospedale Maggiore Policlinico, Mangiagalli e Regina Elena, Milano, Italy; 33 Nephrology, Dialysis, and Organ Transplant Unit, University Hospital, CHU Rangueil, Toulouse, France; 34 Department of Surgery, Saint-Luc Hospital, CHUM, Montreal, Quebec, Canada; 35 Liver Transplant Unit, Hospital Universitario Puerta de Hierro, Madrid, Spain; 36 Department of General Surgery, Ruprecht-Karls-University, Heidelberg, Germany; 37 Department of General & Hepato-biliary Surgery, Liver Transplantation Service, Ghent University Hospital Medical School, Ghent, Belgium; 38 Department of Gastroenterology and Hepatology, Leiden University Medical Centre, Leiden, the Netherlands; 39 Department of Transplantation, San Martino University Hospital, Genoa, Italy; 40 Centre de Chirurgie Viscérale et de Transplantation, Hôpital de Hautepierre, Strasbourg, France; 41 Department of General and Visceral Surgery, Münster University, Münster, Germany; 42 Hepatobiliary Pancreatic Surgery Unit, Queen Elizabeth Hospital, Edgbaston, Birmingham, UK; 43 Surgical Department, Transplant Section, The Rikshospitalet University Hospital, Oslo, Norway; 44 Division for Transplantation, Department of Surgery, Medical University Vienna, Vienna, Austria; 45 Department of Transplantation Surgery, Karolinska University Hospital Huddinge, Stockholm, Sweden; 46 AW Morrow Gastroenterology and Liver Centre, Royal Prince Alfred Hospital, Sydney NSW 2050, Australia; 47 Department of General, Visceral, and Transplantation Surgery, Charité Universitätsmedizin Berlin, Berlin, Germany

## Abstract

**Background:**

The potential anti-cancer effects of mammalian target of rapamycin (mTOR) inhibitors are being intensively studied. To date, however, few randomised clinical trials (RCT) have been performed to demonstrate anti-neoplastic effects in the pure oncology setting, and at present, no oncology endpoint-directed RCT has been reported in the high-malignancy risk population of immunosuppressed transplant recipients. Interestingly, since mTOR inhibitors have both immunosuppressive and anti-cancer effects, they have the potential to simultaneously protect against immunologic graft loss and tumour development. Therefore, we designed a prospective RCT to determine if the mTOR inhibitor sirolimus can improve hepatocellular carcinoma (HCC)-free patient survival in liver transplant (LT) recipients with a pre-transplant diagnosis of HCC.

**Methods/Design:**

The study is an open-labelled, randomised, RCT comparing sirolimus-containing versus mTOR-inhibitor-free immunosuppression in patients undergoing LT for HCC. Patients with a histologically confirmed HCC diagnosis are randomised into 2 groups within 4-6 weeks after LT; one arm is maintained on a centre-specific mTOR-inhibitor-free immunosuppressive protocol and the second arm is maintained on a centre-specific mTOR-inhibitor-free immunosuppressive protocol for the first 4-6 weeks, at which time sirolimus is initiated. A 2^1/2^ -year recruitment phase is planned with a 5-year follow-up, testing HCC-free survival as the primary endpoint. Our hypothesis is that sirolimus use in the second arm of the study will improve HCC-free survival. The study is a non-commercial investigator-initiated trial (IIT) sponsored by the University Hospital Regensburg and is endorsed by the European Liver and Intestine Transplant Association; 13 countries within Europe, Canada and Australia are participating.

**Discussion:**

If our hypothesis is correct that mTOR inhibition can reduce HCC tumour growth while simultaneously providing immunosuppression to protect the liver allograft from rejection, patients should experience less post-transplant problems with HCC recurrence, and therefore could expect a longer and better quality of life. A positive outcome will likely change the standard of posttransplant immunosuppressive care for LT patients with HCC.

**Trial Register:**

Trial registered at http://www.clinicaltrials.gov: NCT00355862

(EudraCT Number: 2005-005362-36)

## Background

Patients with HCC that receive a LT in an attempt to cure their cancer and any superimposed liver disease face at least two major issues. First, the patient requires adequate immunosuppressive medication to avoid rejection of the liver allograft. Second, the patient has a risk that the HCC recurrence could recur, especially when in an immunosuppressed state. Even when restricting LT to patients with limited tumour expansion (e.g. Milan Criteria [1]), some HCCs recur. Furthermore, a significant number of pre-LT analyses of tumour extent are underestimated according to pathologic reports on explanted livers, leaving certain patients at a particularly high risk for HCC recurrence.

Adding to the problem of HCC recurrence, immunosuppressive agents used to prevent allograft rejection are generally regarded as tumourogenic, or at least permissive of cancer development. It is particularly notable that the most commonly used immunosuppressive class of compounds in LT patients, calcineurin inhibitors (cyclosporine and tacrolimus), have been implicated to support tumour formation. Cyclosporine has been shown *in vitro *to enhance cancer cell invasiveness [2] and support angiogenesis accompanying tumours [3,4]. It has also been reported that cyclosporine inhibits DNA repair mechanisms [5], potentially promoting tumour development. Regarding LT, cyclosporine has been shown to promote liver tumour growth and recurrence in an experimental rat model [6]. In other experimental studies, a higher proliferation rate of human hepatoma cells could be demonstrated in the presence of another calcineurin inhibitor, tacrolimus [7]. It remains, however, unproven whether calcineurin inhibitors actually result in a higher HCC recurrence in the setting of LT.

A new view towards this "old problem" of HCC recurrence is supported by recent studies showing that one class of immunosuppressants, mTOR inhibitors, is capable of not only inhibiting immune responses against transplanted allografts, they may also be potent antineoplastic agents. Rapamycin, as the first described mTOR inhibitor, has strong antiangiogenetic effects that inhibit tumour growth in numerous experimental models [3,4,8]. Indirect inhibition of tumor metastasis has also been reportedly due to increased E-cadherin expression on tumour cells [9]. Not only does rapamycin inhibit tumour growth indirectly, cancer cells themselves are inhibited directly by their variable dependence on the mTOR pathway for cell growth and survival [10]. Interestingly, HCC tends to be highly vascularised [11], suggesting a potential susceptibility to rapamycin. Moreover, experimental models indicate that the mTOR signalling pathway is utilised by hepatic tumour cells [12].

From a clinical perspective, mTOR inhibitors have begun to show efficacy with some types of cancer, including especially advanced renal cell carcinoma [13,14]. Little information is available in the context of organ transplantation. While early indications from transplant registry data [15], and from studies not directed at determining tumour development, suggest a general decrease in cancer with mTOR inhibitors, no prospective randomised data has yet confirmed this idea. Most data published with respect to tumour development in transplant recipients has been with the mTOR inhibitor, sirolimus. Although not powered for an oncologic endpoint, studies using sirolimus suggest skin cancer, and other malignancies may be fewer in transplant recipients [16]. Small non-randomised uncontrolled pilot trials and retrospective analyses also hint that sirolimus may improve the outcome for LT patients with a pretransplant diagnosis of HCC [17,18], regarding both tumour recurrence and a more benign course of renewed HCC disease [19]. Unfortunately, these ostensible effects can only be confirmed in a controlled prospective randomised clinical trial.

Based on these experimental and clinical observations, we have designed a clinical trial protocol with the purpose of testing whether the mTOR inhibitor sirolimus can improve HCC-free survival after LT. We predict that patients treated with sirolimus will experience an improved HCC-free survival.

## Methods/Design

### Basic protocol overview

**Figure 1:**
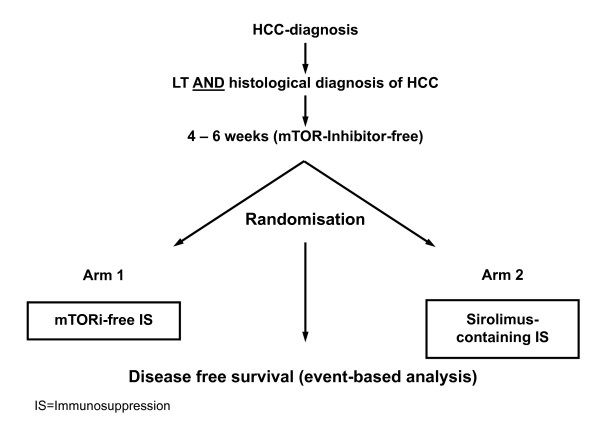
**Inclusion and Randomisation Scheme**.

This is an open-labeled, randomised, prospective multi-center clinical trial comparing sirolimus-containing versus mTOR-inhibitor-free immunosuppression in patients undergoing LT for HCC. We have named the trial the "SiLVER Study", referring to the use of sirolimus in LT patients with HCC. The study is planned for 8 years in total, consisting of a 3-year enrolment period and a 5-year follow-up. Patients with a histologically proven HCC either within Milan Criteria or with extended criteria will be randomised into 2 groups between day 22 and 42 (inclusive) after LT. The first group will be maintained on a centre-specific mTOR-inhibitor-free immunosuppressive protocol. This control arm will be compared to a second group of patients that will be treated with the centre-specific protocol for the first 4-6 weeks, at which time sirolimus will be incorporated (between day 29 and day 42) into the regime either as a monotherapy, or as a combination therapy with non-mTOR-inhibitor-based immunosuppression (Figure 1).

Notably, HCC staging will be assessed in the explanted liver to determine the actual extent of tumor presence. This information will be used with the pre-LT data to determine if the patient is to be stratified into a high or low risk group, based primarily on fulfilment of Milan Criteria.

### Inclusion criteria

The study includes all patients eligible for LT as outlined by each of the participating centres, including deceased whole-allograft and split-liver donors, as well as living donors.

General inclusion criteria are: age over 18 years, histologically proven HCC before randomisation and signed, written informed consent given by the patient. Individual patients who were treated pre-LT for histologically proven HCC, for example by chemoembolisation, radiofrequency ablation, or percutaneous ethanol instillation, may show tumour reduction, or even complete necrosis, after posttransplant histological staging in the explanted liver. If there is complete tumour necrosis, histological confirmation of HCC post-LT will not be possible. In these cases, patients with a pretransplant histological diagnosis of HCC may still be included in the study. However, complete biological tumour response patients will be down-staged and therefore stratified into the group considered within Milan criteria. In all patients with a pre-LT histological proof of HCC, and an incomplete tumour response following pretreatment, stratification will be solely performed according to the final postoperative histology. For example, in the case of pre-LT diagnosis of an HCC outside the Milan criteria, and subsequent post-LT pathology revealing reduced tumour extent within the Milan criteria, final stratification will be performed according to the post-LT histology report.

### Exclusion criteria

Patients with extrahepatic non-HCC malignancies within the past 5 years (excluding successfully treated squamous cell carcinoma and basal cell carcinoma of the skin) will be excluded. Multiple-organ recipients, patients with a known hypersensitivity to sirolimus or its derivates, hyperlipidemia refractory to medical management, evidence of local or systemic infection, HIV, platelets <75,000/nl, women of child-bearing potential not willing to take contraception, patients with extrahepatic HCC tumour manifestation, and patients with a psychological, familial, sociologic or geographic condition potentially hampering compliance with the study protocol and follow-up schedule or under guardianship (e.g. individuals who are not able to freely give their informed consent), and patients receiving mTOR inhibitors prior to day 29 after LT will also be excluded.

### Study objectives

Our hypothesis is that sirolimus use in the test arm of the study will improve HCC-free survival in this LT population. The primary endpoint in this trial is HCC recurrence-free patient survival with sirolimus-containing versus mTOR-inhibitor-free immunosuppression in patients undergoing LT for HCC. Secondary endpoints will be patient overall survival, incidence of *de novo *malignancies, liver allograft and kidney function, HCV and HBV recurrence, time to HCC recurrence, tumour number and size at the time of recurrence, tumour progression rate, and HCC-free, as well as, overall -survival in high and low -risk groups.

### Randomisation and treatment scheme

Patients will be randomised via an interactive voice response system (IVRS) into one of the two treatment arms after histologically proven HCC. After the patient is assigned to a treatment group, a code to uniquely identify the patient (Patient ID) and the assigned treatment group is transmitted via fax. In treatment arm 1, patients are treated with the centre-specific immunosuppressive protocol, excluding the use of any mTOR-inhibitor medications. Steroid reduction is encouraged by 3 months post-LT. In treatment arm 2, application of sirolimus is started between day 29 and 42 after LT with a loading dose of 5 mg/d, and 2 mg/d thereafter. The first sirolimus trough level is measured after 3 to 4 days, followed by trough levels once a week for 4 weeks and twice a month thereafter. The desired trough level for sirolimus is 4-10 ng/ml. Simultaneously, the use of mycophenolic acid prodrugs (mycophenolate mofetil, Roche; enteric-coated mycophenolate sodium, Novartis) and of calcineurin inhibitors should be reduced by 50%. Steroid reduction by 3 months is encouraged. The ideal long term, but not obligate, goal is sirolimus mono-therapy in arm 2.

### Follow-up and documentation

In the first year after LT all patients will be followed-up after month 1, 3, 6, 9 and 12. Thereafter, patients are followed-up every 6 months. Sirolimus levels in patients within arm 2 will be tested and adjusted if need be at each follow-up date.

In general, regular documentation includes data on graft survival, graft dunction as measured by laboratory values, incidence of clinically-diagnosed and biopsy proven acute graft rejection, severity of rejection (histological grade), incidence of premature withdrawal from study medication for any reason, changes in glucose or lipid metabolism, renal function, changes in systolic and/or diastolic blood pressure, adverse events, infectious complications, wound-healing disturbances, haematological toxicity, gastrointestinal side-effects, thrombembolic complications, tumour recurrence information and patient survival.

If a patient should miss two consecutive follow-up visits and the investigator cannot establish contact with the patient, the status of the patient will be set to 'lost-to-follow-up'. If the last contact was prior to the diagnosis of a recurrence event, recurrence-free survival time will be censored using the date of the last contact. If contact with the patient can be re-established, the status for survival time will be reset to alive. However, recurrence-free survival time and accompanying censoring status will remain. If the date of a patient's death is documented, the status will be "dead".

### Endpoint definition

HCC recurrence-free survival (RFS) is specifically defined as the time interval between the date of LT and the date of HCC recurrence or death (as first event); patients who are alive and recurrence-free at the time of analysis will be censored for RFS at the time of their last contact. HCC recurrence is defined as either histologically-proven tumour recurrence, or unequivocal tumor recurrence determined by the Barcelona Criteria (Barcelona-2000 EASL Conference). Date of recurrence is defined as the first day of tumor suspicion. Clinical evidence for HCC recurrence including β-symptoms, weight loss, inappetence, pruritis, and ascites are considered suspicious signs and are followed up by definitive diagnostic action in accordance with the Barcelona Criteria. Recurrence of HCC can therefore be determined during the entire follow-up period, and not only at protocol-assigned visits.

### Trial organization

The SiLVER Study is an investigator-initiated trial. Preliminary investigator meetings to plan the specifics of the trial protocol were organised by the Department of Surgery, University of Regensburg (University Hospital Regensburg). Indeed, University Hospital Regensburg is the sponsor of the study. Funding aid is through a grant provided by Pfizer (Collegeville, PA, USA). Contract research organisation (CRO) services for site monitoring and regulatory affairs are provided by Chiltern International, Bad Homburg, Germany. The eCRF and IVRS-services are provided by ClinIT, Freiburg, Germany. Drug storage, re-labelling and distribution tasks are outsourced to B&C Clinipack, Wavre, Belgium. Statistical research was performed within the Department of Surgery, University Hospital Regensburg, and the statistical plan was developed together with the Regensburg Center for Clinical Studies.

### Participating Centers

Major liver transplant centres from Europe, Australia and Canada are participating. In total, 45 sites from 13 countries (Germany, Austria, France, Spain, Italy, Belgium, Netherlands, UK, Sweden, Norway, Finland, Australia and Canada) have committed to the study. Multiple centres were required for this study, since enrolling the planned number of LT patients with HCC could only be accomplished through a broad-based concerted effort.

### Drug supply

One mg and 2 mg sirolimus (Rapamune) blisters, as well as a single 5 mg starting dose pack, is supplied as study medication to the participating centres via B&C Clinipack.

### On-site monitoring

During recruitment and follow-up of patients, regular monitoring of safety and endpoint data is performed according to good clinical practice (GCP) guidelines. Data management is performed by ClinIT and by central monitoring at the sponsoring institution.

### Ethics and safety

Most recently, Protocol Version 9 has been approved by the responsible national/local ethics board for each of the sites participating in the study. We have recently described the ethical review process for our study, in detail [20]. The study protocol has been approved by ethics committees serving the following institutions: (Germany) University Hospital Regensburg, University Hospital of Schleswig-Holstein Campus Kiel, University Leipzig, University Hospital Tübingen, Charité Universitätsmedizin Berlin, Munich University Grosshadern Campus, University Hospital Essen, Johann Wolfgang Goethe University Frankfurt, Medizinische Hochschule Hannover, Ruprecht-Karls-University Heidelberg, Johannes Gutenberg University Mainz Hospital, Münster University, University Hospital of the Friedrich-Schiller-University Jena; (Austria) Medical University Innsbruck, Medical University Vienna; (Spain) University Hospital Puerta da Hierro Madrid, University Hospital Vall d'Hebron Barcelona; (Sweden) Karolinska University Hospital Huddinge Stockholm, The Rikshospitalet University Hospital Oslo; (Netherlands) Leiden University Medical Centre, University Medical Center Groningen; (Belgium) Ghent University Hospital Medical School, University Hospital Leuven, Université Catholique de Louvain Brussels; (Italy) University Hospital Bologna, Azienda Ospedaliera "Ospedali Riuniti" Bergamo, University Hospital of Padua, San Martino University Hospital Genova, National Cancer Institute Milan, Fondazione IRCCS Ospedale Maggiore Policlinico Mangiagalli e Regina Elena Milano; (Finland) Helsinki University Hospital; (Canada) University of Alberta Edmonton, University of Montreal; (Australia) Royal Prince Alfred Hospital Camperdown NSW Sydney. National central ethics commissions in Great Britain (Cambridgeshire Research Ethics Committee - formerly Eastern Multi Center Research Ethics Committee) and France (the Consultative Committee for the Protection of People in Biomedical Research CCPPRB Créteil- Henri Mondor) approved the study protocol for the participating centres in these two countries.

The study complies with the Declaration of Helsinki and the principles of Good Clinical Practice (GCP) guidelines. Informed consent is obtained from each patient in written form prior to randomisation. The patient is informed about the nature, duration and possible consequences of the trial by a medical doctor familiar with the study. Patient safety and all potential threats for the patients are being monitored once a year by an independent data safety monitoring board (DSMB), or additionally at the discretion of the DSMB or Sponsor; the DSMB also will confidentially evaluate the primary endpoint data. Qualified personnel at sponsor site also meet every three months to review safety data, including adverse events (AE) and serious adverse events (SAE). Any information deemed to potentially affect the safety of the trial will be brought to the attention of the DSMB.

### Sample size

Sample size was calculated using the primary endpoint of RFS assuming proportional hazards and exponential distributions of RFS. RFS time distributions of combined high and low risk patients in the two treatment groups will be compared using a two-sided (stratified) log-rank test at a 0.05 significance level. A 5-year RFS rate of 60% (or equivalently an event rate of 40%) in patients treated with mTOR-inhibitor-free immunosuppression is expected. An increase to a 5-year RFS rate of 72% (or equivalently a decrease to an event rate of 28%) due to sirolimus-containing immunosuppression is assumed. The improvement in 5-year RFS rate from 60% to 72% (or equivalently a decrease in 5-year recurrence event rate from 40% to 28%) corresponds to a hazard ratio of 0.643 and is considered as clinically relevant. For detecting a HR of 0.643 with a power of 1-β = 0.80 in the three-stage group sequential design with an α spending function of the O'Brien and Fleming type, it is necessary to observe 164 events (HCC recurrences or deaths). Assuming an accrual time of 2 1/2 years and a follow-up time of at least 5 years from the last patient recruited, a total of 405 patients are expected to yield the necessary number of events. With a lost to follow-up rate of about 20%, a total of 510 patients (255 per treatment group) are required.

### Statistical evaluation

The problem is statistically formulated as a test of the null hypothesis H_0_: θ = 1 versus the alternative hypothesis H_1_: θ ≠ 1, where the hazard ratio (HR) is defined as the risk of recurrence in the sirolimus-containing immunosuppression group divided by the risk of recurrence in the mTOR-inhibitor-free immunosuppression group. Rejection of the above null hypothesis suggests that there is a statistically significant difference between the mTOR-inhibitor-free immunosuppression and the sirolimus-containing immunosuppression. An HR of less than 1 indicates superiority of sirolimus-containing immunosuppression.

RFS distribution and median RFS time will be estimated for the two treatment arms using the Kaplan-Meier method. The two-sided, (stratified) log-rank test will be applied to test the RFS time null hypothesis assuming proportional hazard rates at a 0.05 significance level. Primary analysis will be based on the intention-to-treat analysis set, however, to assess the robustness of the results, a sensitivity analysis will be conducted on a per protocol analysis set. Interim analyses will be performed for ethical and practical reasons. The confirmatory analysis of the primary endpoint will be done using a group sequential analysis plan that is based on the number of observed events. A design with a maximum of three stages was chosen, whereby two interim analyses followed by one final analysis are planned after 55, 109, and 164 events, respectively.

Secondary endpoints will be analyzed in purely an exploratory manner. As part of this secondary analysis, we will compare RFS in two cohorts of patients with different disease-free and overall survival probabilities. Patients with HCC and cirrhosis within the Milan criteria will be defined as "low-risk" patients, whereas patients with HCC extending beyond Milan criteria, patients undergoing salvage transplantation or an HCC in non-cirrhotic liver, will be defined as "high-risk".

## Discussion

Hepatic cancer recurrence continues to be a serious consideration in patients receiving a LT for HCC. Early 2003 ELTR data showed a 5-year patient overall survival for hepatic malignancy (primarily HCC) of merely 53%, comparing poorly with data from non-cholestatic liver cirrhosis of 74% and even acute liver failure of 62%. Since the landmark publication in 1996 by Mazzaferro et al., many centres have restricted their indication for HCC-related LT due to clinical criteria based on tumour size and number ('Milan Criteria'), and other similar systems have since been used [21]. With these improved criteria, single-centre data do indeed show a significant improvement in both disease-free and overall survival following LT for HCC. Nonetheless, recurrences of HCC are not eliminated even with restricting tumour size and number, and importantly, pre-transplant evaluations of tumour extent many times are underestimated, leading to a risk for HCC recurrence in this transplanted subpopulation. Considering the persistence of this problem, and intriguing new data that mTOR inhibitors at immunosuppressive doses have potential anticancer effects, we took the initiative to rigorously test the hypothesis that introduction of the mTOR inhibitor sirolimus could further improve disease-free survival in typical LT patients with a history of HCC.

Among the important considerations when designing this trial was the inclusion of both patients with a low and high risk for HCC recurrence. After much debate, our study group concluded that all patients that would normally be transplanted should be included into the study. With this practical approach, we could answer the question whether the typical HCC group eligible for LT, as a whole (including both low and later discovered high risk patients), would benefit from an immunosuppressive therapy containing sirolimus. Deciding to primarily analyse HCC RFS was another critical point. Our reasoning for this choice was that it could be possible to decrease HCC recurrence with sirolimus, but if other unforeseen complications of this therapy were eventually found to reduce 5-year survival, in the end there would be no overall benefit to the patient; clearly, our study aims for an overall benefit to the patient on the long-term. Finally, we would also like to add that our protocol design is aimed for practical implementation. Essentially, we will compare a "non-mTOR inhibitor" containing immunosuppressive regimen (arm 1) to a regimen that contains the mTOR inhibitor sirolimus. Our approach considers the wide-ranging world-wide opinions from centre-to-centre regarding proper and safe immunosuppression following LT. Each centre is basically allowed to use their standard regimen, as long as mTOR inhibitors are not used in arm 1 and sirolimus is used in arm 2, on an intention-to-treat basis. Experimental work [3], and registry data [15], support the contention that mTOR inhibitors maintain their anticancer effects even in the presence of other immunosuppressants.

The SiLVER Study is the first prospective randomised controlled international trial which evaluates HCC recurrence in patients normally eligible for LT. If our hypothesis is correct that mTOR inhibition with sirolimus can improve RFS, while simultaneously providing adequate immunosuppression, patients with HCC that receive a LT could expect a longer and better quality of life.

## Competing interests

AAS receives clinical trial funding from Roche and Novartis. EKG and HJS, as well as other authors, have received speaking honoraria from Novartis, Roche, Wyeth, Genzyme and Astellas pharmaceutical companies. EKG has received a grant from Wyeth to support the sponsor (University Hospital Regensburg) in the conduct of this trial.

## Authors' contributions

AAS contributed to the study design, coordination, ethics approval processes and wrote the paper. CZ helped with study design and coordination. JR is the study statistician. CG, IB, PB, KPdeJ, CD, KK, RA, WOB, TB, SB, OC, UDdiC, MC, FF, JG, JPH, MH, EH, NJ, AK, PEL, JPL, HM, RM, VM, IM, GO, GPP, ADP, JP, MR, GR, LR, AR, VST, JS, RT, BvanH, UV, PW, HHW, DFM, TS, RS, GS, SIS, KWJ, and PN participated in study design and local ethics approval. HJS was involved with study design, ethics approval and medical monitoring. EKG initiated the study concept, design, coordination, ethics approval and wrote the paper. All authors read and approved the final manuscript.

## Pre-publication history

The pre-publication history for this paper can be accessed here:

http://www.biomedcentral.com/1471-2407/10/190/prepub
